# Sensing Precursors of Illegal Drugs—Rapid Detection of Acetic Anhydride Vapors at Trace Levels Using Photoionization Detection and Ion Mobility Spectrometry

**DOI:** 10.3390/molecules25081852

**Published:** 2020-04-17

**Authors:** Victor Bocos-Bintintan, George-Bogdan Ghira, Mircea Anton, Aurel-Vasile Martiniuc, Ileana-Andreea Ratiu

**Affiliations:** 1 Faculty of Environmental Science and Engineering, Babeş-Bolyai University, Str. Fântânele nr. 30, RO-400294 Cluj-Napoca, Romania; ghira_george@yahoo.com (G.-B.G.); mirceaanton@yahoo.com (M.A.); 2 Technische Universität München, Institut für Informatik VI, Boltzmannstraße 3, 85748 Garching bei München, Germany; aurelmartiniuc@gmail.com; 3 Babeș-Bolyai University, “Raluca Ripan” Institute for Research in Chemistry, 30 Fântânele Str., RO-400294 Cluj-Napoca, Romania; 4 Department of Environmental Chemistry and Bioanalytics, Faculty of Chemistry, Interdisciplinary Centre of Modern Technologies, Nicolaus Copernicus University, 7 Gagarina Str., 87-100 Torun, Poland

**Keywords:** acetic anhydride (AA), trace detection, illegal drugs precursors, photoionization detection PID, ion mobility spectrometry IMS

## Abstract

Sensitive real-time detection of vapors produced by the precursors, reagents and solvents used in the illegal drugs manufacture represents a priority nowadays. Acetic anhydride (AA) is the key chemical used as acetylation agent in producing the illegal drugs heroin and methaqualone. This study was directed towards quick detection and quantification of AA in air, using two fast and very sensitive analytical techniques: photoionization detection (PID) and ion mobility spectrometry (IMS). Results obtained indicated that both PID and IMS can sense AA at ultra-trace levels in air, but while PID produces a non-selective response, IMS offers richer information. Ion mobility spectrometric response in the positive ion mode presented one product ion, at reduced ion mobility K_0_ of 1.89 cm^2^ V^−1^ s^−1^ (almost overlapped with positive reactant ion peak), while in the negative ion mode two well separated product ions, with K_0_ of 1.90 and 1.71 cm^2^ V^−1^ s^−1^, were noticed. Our study showed that by using a portable, commercial IMS system (model Mini IMS, I.U.T. GmbH Berlin) AA can be easily measured at concentrations of 0.05 ppm_v_ (0.2 mg m^−3^) in negative ion mode. Best selectivity and sensitivity of the IMS response were therefore achieved in the negative operation mode.

## 1. Introduction

Illegal drugs are currently detected in a direct way, in real time, using commercial trace detectors, most of them based on ion mobility spectrometry (IMS); these IMS detectors are widely used worldwide, e.g., in all major airports, in governmental buildings, customs and so forth. However, these instruments are quite bulky and have large power requirements. In addition, detection of illicit drugs is a difficult process since these compounds have very low vapor pressures, which necessitates usually a sampling step by collecting solid particles, followed by the vaporization of the collected micro-particles of drugs by flash thermal desorption before the analysis by IMS [1,2]. 

Ion mobility spectrometry (IMS) has been successfully developed to yield advanced portable instrumentation. Such instruments are able to quickly detect trace quantities of controlled substances on site or at the crime scene—such as traces of explosives, illegal drugs, chemical and biological warfare agents, toxic chemicals or bacterial markers [1,3,4,5,6,7,8,9,10,11]. IMS is also routinely and extensively used by customs and law enforcement agencies (e.g., in international airports) for the detection of illegal drugs (by monitoring positive ions) and explosives (by monitoring negative ions). Thousands of IMS devices detecting drugs and explosives at ultra-trace levels are already deployed worldwide, most of them in airports. The main advantages of IMS are: simple and rapid sampling; very high sensitivity, and compact, robust and miniaturized instrumentation [4,5,6]. 

A much faster and simpler alternative to the standard, direct drug detection procedure described above is to indirectly sense the illegal drugs by detecting those chemicals that are closely associated to them—such as the precursors, reagents and solvents used in the manufacture process—because most of these chemicals have a higher volatility (larger vapor pressure) compared to illegal drugs [7]. For instance, acetic anhydride is a key chemical used as an acetylation agent in manufacturing heroin (from morphine) and methaqualone; therefore, by detecting acetic anhydride at trace levels (<ppm_v_) in the air of a room, if any licit use of this compound is rejected, then its presence may be indicative of clandestine chemical processes, including the manufacture of heroin within that building. Same considerations may apply for air samples from a transport container.

Illicit drug manufacture of heroin is a serious, continuously growing, international problem. The reason for including acetic anhydride (AA) in the Table 1 of scheduled controlled substances is exactly its use, by drug manufacturers, as an acetylation agent in heroin production [12]. Millions of liters of AA are traded annually worldwide, whereby only a small fraction is required for drug manufacture. According to the US State Department, from the total amount of AA available for illicit heroin manufacturing, only a small percent is seized annually by law enforcement agencies—between 2 and 8%, leaving on the illicit market an estimated quantity between 700,000 and 2,600,000 L of AA for heroin production [13,14]. For clandestine production of heroin, acetic anhydride AA is a common precursor/reagent, being used as an acetylation agent for morphine, from which yields diacetylmorphine (heroin). Chemical and physical properties of AA are listed in Table 1.

Although not a major producer of illicit drugs, Romania still remains a major transit country for narcotics, since it lies along the Northern Balkan Route for heroin that moves from Afghanistan to Central and Western Europe [14]. In 2010, the Romanian authorities made a total of 962 heroin seizures, summing 108 kg of heroin. In the same year there were 33 drug-related deaths, wherefrom 78.78% were caused by opiates consumption and 53% from opiates where caused by heroin/morphine [13]. In 2011, according to DIICOT (the Romanian anti-organized crime division), a total of 12.19 kg of heroin were seized [15,16]. Several studies regarding the relationship between different heroin seizures were conducted and statistical analysis based on comparison correlation of coefficient provides a tool for law enforcement in linking the seizures; moreover, slight variation in sample composition does not affect the forecasting nature of the results [17,18]. 

From the toxicological point of view, AA can function as a hapten that may bind to proteins in the respiratory tract and then elicit an immune response. After this sensitization, the subsequent exposures to AA could easily lead to asthma-like symptoms. Furthermore, chronic exposure may produce severe restrictive lung diseases [19]. Exposure limits for AA, as given by the American Occupational Safety and Health Administration (OSHA) and National Institute for Occupational Safety and Health (NIOSH), are both a Permissible Exposure Limit (PEL) and Time Weighted Average (TWA) of 5 ppm_v_ (ca. 21 mg m^−3^). The Immediate Danger for Life and Health (IDLH) level has been estimated to be at 200 ppm_v_ (850 mg m^−3^) by National Institute for Occupational Safety and Health (NIOSH, USA). Exposure to AA will cause severe irritation of the eyes, skin and mucous membranes in humans. Being a widely used acetylation agent, AA is used in both licit and illicit activities. For example, licit uses of AA include: wood preservation, which results in a more durable timber [20]; obtaining acetylated corn and potato starches used in food industry [21]; as acetylating and dehydrating agent in chemical and pharmaceutical industries [13]. On the other hand, illicit uses of AA are related to heroin and methaqualone production. 

This study was directed towards quick detection and quantification of acetic anhydride in indoor air, using two analytical techniques that are both rapid and very sensitive: photoionization detection (PID) and ion mobility spectrometry (IMS). 

Detection of AA was also reported by using other classical analytical techniques, such as gas or liquid chromatography coupled with mass spectrometry [22]. Moreover, dedicated sensors for real time detection of AA were manufactured; for instance, Nugroho and co-authors reported the development of new nanofibers from cellulose acetate and chitosan used for the detection of AA [23]. The obtained results highlighted a fast response (44 s) and decently good sensitivity (10–1000 ppm), with a reported lower limit of detection of 5 ppm [22]. However, these figures of merit are nevertheless poorer than the sensitivity and speed of response of both IMS (<0.1 ppm_v_) and PID (low ppb_v_) devices which were used during our investigation. 

Our study showed that by using a portable, commercial IMS system (model Mini-IMS, made by I.U.T. GmbH Berlin, Germany), acetic anhydride can be easily sensed at concentrations as low as 50 ppb_v_ in negative ion mode, and at ppm_v_ levels in positive ion mode. Fast sensing of AA vapors has a paramount importance, because this chemical is a controlled substance that may be used for manufacturing illegal drugs. In addition, its quantitation has great impact in protecting the health of the law-enforcement personnel that investigate a clandestine drug production facility.

## 2. Results and Discussion

Experimental data generated by the photoionization detector (PID) were gathered simultaneously with the data produced by the ion mobility spectrometer (IMS). Therefore, we may claim that PID readings were indicating the actual, real concentration of AA vapors in the standard atmospheres generated inside the 10 L glass flask. 

Ion mobility spectrometric responses from AA were obtained in both negative and positive ion modes, which constitute a real advantage for the identification of a target chemical—because very few compounds produce IMS responses in both ion modes. However, it can be reasonably assume that the response produced in the negative ion mode is related to a hydrolysis reaction of acetic anhydride that leads into the formation of acetic acid.

IMS response resulted in simple spectra, where two product ion peaks (PIPs) can be assigned to AA in the negative ion mode and one PIP in the positive mode. The positive reactant ion peak (RIP) was observed at a drift time t_d_ = 6.28 ms, while the negative reactant ion peak (RIP) was noticed at a drift time t_d_ = 5.98 ms.

**Figure 1:**
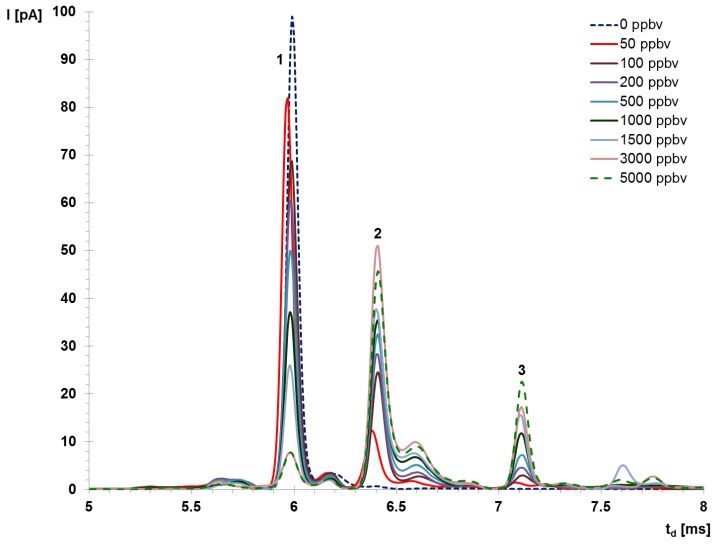
Ion mobility spectrometry (IMS) response to acetic anhydride (AA) in the negative mode, where: 1—RIP; 2—PIP#1; 3—PIP#2. Note: although the ion mobility spectra are collected from 1 to 20 ms, in order to increase the clarity only the useful part of the spectra, namely the temporal interval that includes all peaks, has been showed.

As an example, the IMS spectra obtained in the negative mode are presented in Figure 1 and are clearly highlighting the change in the intensity of the peaks with the increase of the AA measured concentration. When AA vapor concentration increases, the intensity of the negative RIP decreases, while the heights of PIP#1 and PIP#2 increase. The peak observed at a t_d_ of ca. 7.6 ms is most probably generated by impurities since it remained relatively constant when the AA concentration changed.

The nature of the ions produced in the IMS cell can be investigated and then assigned with a high degree of certainty only by coupling a mass spectrometer to the IMS instrument, which means using a hyphenated, complex analytical system IMS-MS. IMS-MS devices were used mainly in order to investigate the identity of ions generated by the highly toxic chemicals, such as for instance chlorine and phosgene [4,5]. Assigning the ions generated inside the IMS cell by AA was not feasible, since in the present study it was utilized just an IMS device. It can only make the assumption that, for instance, in the negative ion mode the AA generated two product ions: PIP#1, which may be regarded as a monomer (an ion that includes one molecule of analyte), and PIP#2, which could be considered as a dimer (containing two molecules of analyte).

The combined results from both ionization-based analytical techniques (PID and IMS, respectively) are summarized in Table 2. This table is showing in a logical and clear manner the steps and values obtained. Here, C_AA_ measured with PID is the concentration of AA indicated by the ppbRAE Plus photoionization instrument (calculated as the direct PID reading in units of iso-butene multiplied by the correction factor for AA, which is CF_AA_ 6.1 for a 10.6 eV UV lamp). The concentration of AA vapors has been checked using the ppbRAE Plus PID device. Thus, the injection of a small amount of pure liquid AA has the unique role of creating an initial standard atmosphere which, by being diluted using clean air, will further produce a range of standard atmospheres with lower AA vapor concentrations.

**Figure 2:**
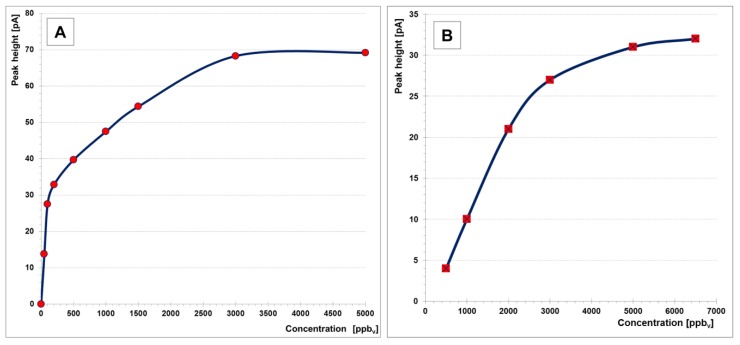
Calibration graphs for AA in the negative (**A**) and positive mode (**B**), normalized for the background air. In the positive ion mode, peak height of the single product ion peak has been plotted; in the negative ion mode, the sum of PIP#1 and PIP#2 heights has been used.

Quantitative data were plotted in order to obtain calibration curves, and finally to evaluate the quantitative response of the ion mobility spectrometer to AA. The calibration graphs are presented in Figure 2A (negative mode) and Figure 2B (positive mode). 

A careful scrutiny of Figure 2 and of ion mobility spectra obtained for all the concentration levels allows us to conclude that:

*1.*  The saturation threshold of the IMS instrument (indicated by the total disappearance of the reactant ion peak RIP) was not reached. This means that the quantitative data provided by the IMS device is reliable and also that contamination of the IMS cell has been successfully avoided.
*2.*  The most sensitive and selective operation mode is the negative ion mode. Apparition of 2 product ion peaks is an additional advantage, since the identification of AA is better by having two peaks to rely on. Minimum concentration measured was 50 ppb_v_ (with both PIP noticed); the estimated minimum concentration level MCL is thought to be ca. 10 ppb_v_ (42 μg m*^−^*^3^), when just the PIP#1 (K_0_ = 1.90 cm^2^ V*^−^*^1^ s*^−^*^1^) is to be seen.
*3.*  In the positive ion mode, one product ion peak was observed, and only at relatively high AA vapor concentrations (>500 ppb_v_) compared to negative mode. This product ion peak is poorly resolved, being practically almost overlapped with the positive RIP; in order to resolve the RIP and the PIP a deconvolution step is highly recommended. For positive ion mode, the estimated minimum concentration level is ca. 500 ppb_v_ AA.
*4.*  Saturation occurs at around 3 ppm_v_ in the negative ion mode, which is in accordance with the well-known fact that the dynamic range of an IMS device with a radioactive source ranges typically on two orders of magnitude. In the positive ion mode, saturation is thought to appear at AA vapor concentrations >6 ppm_v_.
*5.*  Acetic anhydride produced ion mobility spectra in both positive and negative ion mode, which is definitely an advantage from a qualitative identification by IMS. However, negative operation mode is certainly the most advantageous, as explained previously.

The logarithmic aspect of the calibration trend lines is absolutely characteristic for a typical IMS response using a radioactive ionization source [1,2]. In the negative ion mode, the spectra for high AA concentrations (>2000 ppb_v_) indicate that the saturation threshold was still not reached, but it is quite close. Thus, ion mobility spectra at maximum AA levels investigated (of 5000 ppb_v_ or 21.2 mg m^−3^) indicate that saturation has been reached, because the reactant ions has decreased to less than 10%–15% of their initial value, when the AA concentration was 0. In other words, when the saturation is reached the whole amount of reactant ions (positive or negative) has been consumed and the reactant ion peak RIP disappears from the IMS spectrum. Saturation of any IMS instrument must be avoided, because it leads to a heavy contamination of the IMS measurement cell and to subsequent unwanted memory effects.

Consequently, IMS spectra are able to offer both qualitative information (through the specific drift time, and hence the reduced mobility of an ion) and quantitative information (through peak area or peak height). Drift time is proportional to the charge and inversely proportional to the mass and size of ionic clusters. In other words, an ion mobility peak in the spectrum may be characterized by three values: (1) drift time t_d_ (expressed in milliseconds, ms); (2) reduced mobility K_0_ (expressed in cm^2^V^−1^cm^−1^) and (3) maximal amplitude (height) of the peak, h_max_ (expressed in pA).

With respect to Table 3, the first product ion peak PIP can be assigned to the monomer ion (containing just one molecule of AA), while the second PIP, with the lowest ion mobility, could be considered as a dimer ion (which includes two molecules of AA). 

The possible interferences are always a concern. However, we have to emphasize at this point that only a small fraction of chemicals (ca. 20%) generate product ions in the negative ion mode of IMS. Therefore, by using the negative operation mode, practically an ionization-based selectivity is realized; this fact means that there is a much lower probability of possible interfering compounds in the negative ion mode, as compared to using the more prone to interferences positive ion mode.

As concluding remarks, we emphasize that acetic anhydride vapors in air were successfully determined using IMS at trace and ultra-trace levels, between 50 ppb_v_ and 5000 ppb_v_ in the negative ion mode. Detection of AA was successfully accomplished by both PID and IMS. Using IMS, both positive and negative responses were observed, but the most advantageous is by far the negative ion mode, where two distinct product ions, with K_0_ of 1.90 and 1.71 cm^2^ V^−1^ s^−1^, were observed. In other words, best selectivity and sensitivity of the ion mobility spectrometric response were achieved in the negative ion mode. Ion mobility spectrometric response in the positive ion mode implied the existence of one product ion, at reduced ion mobilities of 1.89 cm^2^ V^−1^ s^−1^, which is practically not resolved of positive RIP. The presence of two product ions, very well separated, in the negative ion mode may definitely be regarded as a serious advantage in detecting AA at trace levels, because the IMS device can be set to sense AA using two time intervals in the negative mode, corresponding to the two product ion peaks.

From the quantitative point of view, the concentration range that was explored (from 50 to 5000 ppb_v_, in the negative ion mode) was able to deliver useful information concerning the measurement range before saturation and also to notice that saturation starts at ca. 3 ppm_v_ AA. A minimum detectable concentration MDC of about 10 ppb_v_ (0.042 mg m^−3^) AA, in the negative ion mode, is also estimated. By applying the developed method, 50 ppb_v_ (0.2 mg m^−3^) of AA in air was measured, a concentration that is 100 times lower than the TWA and PEL values (5 ppm_v_) and 4000 times lower than IDLH value. 

The IUT ion mobility spectrometer Mini-IMS has a very good sensitivity for AA present in air samples and may be very useful in real-life situations. Of course, the IMS instrument must be evaluated in real-life scenarios; this could be the object of a future study. IMS instrumentation proves to be an invaluable tool in: (a) quickly detecting AA as an illegal drug precursor or as an impurity present in illicit drugs, and (b) protecting the health of law enforcement officers that investigate the crime scene of a clandestine laboratory.

### Validation

In order to assess the suitability of the developed analytical method to its purpose, validation process was carried out. The following parameters were evaluated: sensitivity, limit of detection and limit of quantitation, range of linear response, accuracy and trueness of the method proposed.

Limit of detection (LOD) was defined as the lowest concentration that provides a signal-to-noise (S/N) ratio equal to 3, while limit of quantitation (LOQ) was defined as the lowest concentration generating an S/N ratio of 10. Sensitivity S is defined as the change in signal Y (namely, peak height) on modification of concentration (S = ΔY/ΔC). The background signal—defined as the standard deviation of the background noise—has been obtained from the first 400 data points (signals from 1.00 to 5.00 milliseconds, in increments of 0.01 millisecond) for every IMS spectrum; the average value has found to be s = 0.104 pA in the negative ion mode and s = 0.218 pA in the positive ion mode, respectively. Table 4 presents the figures of merit related to AA detection.

In the negative ion mode sensitivity, LOD, LOQ and linear range are much better than in the positive ion mode.

Precision was evaluated using analyses in triplicate (see Table 2). Accuracy was assessed by using the relative standard deviation RSD (called also coefficient of variation CV), which was between 3.3% and 6.7% for the PIP#1 in the negative ion mode, and between 2.0% and 3.4% in the positive ion mode. Good repeatability of results was observed, with RSD <10%.

Traceability to a standard reference material SRM—namely the standard of 10 ppm_v_ i-butene in air, used for calibrating the PID instrument—has been accomplished. Note that PID instrument has been used to verify the AA concentrations when they were simultaneously determined using the IMS instrument. Therefore, the use of this Certified Reference Material (CRM) is supporting the trueness of the obtained results.

## 3. Materials and Methods

AA was purchased from Sigma-Aldrich (puriss. p.a., ACS reagent, ≥99.0%) and was used as is, without any purification.

**Figure 3:**
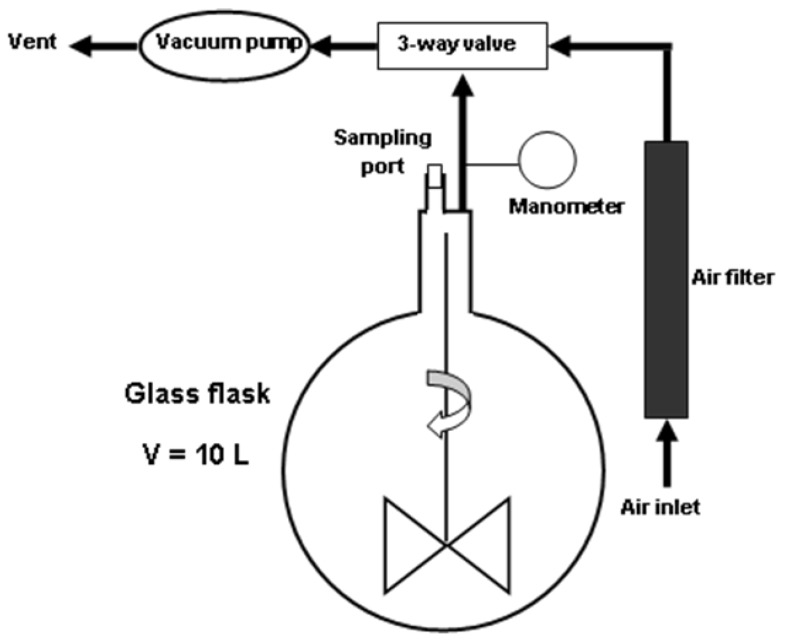
Schematic of the static mixing system used as a TAG.

Standard atmospheres with known low concentrations of target analyte were prepared in a custom-built Test Atmosphere Generator (TAG) (see Figure 3), using a static method based on injection of pure liquid AA: the air from a 10 L glass flask was first evacuated using an oil vacuum pump until a low pressure of ca. 150 mm Hg was reached, then a very small volume of pure, liquid chemical was injected into the flask through a PTFE-silicone septum affixed into the sampling port, by using a 1 µL chromatographic syringe. After about 10 s, the syringe needle was removed from the sampling port and air was allowed to fill up the glass flask. A homogenization system based upon a magnetic stirrer that propelled a fan-type piece placed inside the glass flask has been used. In order to avoid the overloading the IMS instrument with the analyte studied, only a volume less than 1 µL of pure, liquid AA was injected into the glass flask; thus, it was ensured low concentrations of AA vapors in the flask, in the low ppm_v_ range.

Lower concentrations of AA vapors, in the ppb_v_ range, were obtained further by diluting the initial standard atmospheres using purified air.

A range of standard atmospheres with various concentrations of AA vapors in air was obtained, as follows:

- By injecting a volume of 0.04 µL liquid pure AA in the 10 L glass flask, a theoretical concentration C_0 (calculated)_ = 1000 ppb_v_, while the actual/real value was only 500 ppb_v_ AA (82 ppb_v_ i-Butene). Starting from this initial concentration, standard atmospheres with 200, 100 and 50 ppb_v_ AA were further obtained.
- By injecting a volume of 0.20 µL liquid pure AA in the 10 L glass flask, a theoretical concentration C_0 (calculated)_ = 5,100 ppb_v_, while the real value (measured using the PID) was only 2400 ppb_v_ AA (395 ppb_v_ i-Butene). From this initial concentration, standard atmospheres with 2000, 1000 and 500 ppb_v_ AA were generated.
- By injecting a volume of 0.50 µL liquid pure AA in the 10 L glass flask, a theoretical concentration C_0 (calculated)_ = 12,750 ppb_v_, while the real value was 6500 ppb_v_ AA (1065 ppb_v_ i-Butene). From this initial concentration, standard atmospheres with 5000, 4000 and 3000 ppb_v_ AA were produced.

The discrepancy between the calculated initial AA concentration and the real concentration measured with the ppbRAE Plus PID detector could be explained by adsorption, absorption and chemical reactions of AA vapors onto/with the internal parts of the TAG (especially the glass flask and the internal stirring system).

### 3.1. PID Instrument Used

Photoionization detector used was a ppbRAE Plus (Model PGM-7240), manufactured by RAE Systems Inc., Sunnyvale, CA, USA. This is an extremely sensitive photo-ionization detector (PID) for real-time monitoring (with a response time T_90_ < 5 sec) of volatile photoionizable compounds at ppb_v_ levels. With its highly compact design (size 21.8 × 7.6 × 5.0 cm and weighing only 553 g), this volatile organic compounds detector is widely used as an ultrasensitive VOC monitor, having a measurement range from 1 ppb_v_ to 250,000 ppb_v_. The PID sensor has an improved sensitivity at ppb level and uses a standard 10.6 eV UV lamp. The instrument was calibrated prior to analysis with a supplied isobutene (i-C_4_H_8_) calibration gas (gas cylinder, RAE Systems, Inc.), with a concentration of 10 ppm_v_ isobutene in clean air. The built-in sampling pump has an internal flow of 400 cm^3^ min^−1^ and the instrument may be operated between −10 and +40 °C. Data logged into the internal microprocessor can easily be transferred to the PC computer using a COM connection cable and the associated software ProRAE Suite, ver. 3.01a, 2004 [24,25,26,27].

### 3.2. IMS Instrument Used

A portable ion mobility spectrometer model Mini-IMS, produced by the German company I.U.T. GmbH Berlin was used. The most relevant specifications of this instrument are presented in Table 5 [7,28].

A small volume of air sample containing the target analyte (AA) is periodically taken and introduced into the IMS system, where a radioactive source ionizes the molecules in the sample in a soft manner (meaning that analyte molecules are not fragmented, as it occurs in electron-ionization mass spectrometry EI-MS). After the formation of product ions (ions that include the whole molecule of target analyte), they drift towards detector under the influence of an electric d.c. field E, traveling with a constant speed (several m s^−1^) inside the so-called “drift cell“. When the ions reach the end of the drift cell and arrive at the detector, they generate a low ion current (pA range), which is further amplified and measured. Each compound has a typical drift velocity in the drift gas and may therefore be identified using the drift time [1]. The physical principle of IMS is thus based on ions’ separation, based on the different drift velocities of either positive or negative ions in a homogeneous electric field, at atmospheric pressure. The air sample arrives at an ion source and is ionized there by a radioactive source, generating the so-called “reactant ions”, which are complex cluster ions like (H_2_O)_n_H^+^ (these hydrated protons are the vast majority), plus (H_2_O)_n_NH_4_^+^ and (H_2_O)_n_NO^+^ (in the positive ion mode), or (H_2_O)_n_O_2_^-^ (in the negative ion mode) [1,2]. This is a soft ionization (since the molecule of analyte is not fragmented) that occurs in two steps—formation of reactant ions, followed by generation of product ions by collisional charge transfer from reactant ions towards the analyte. This ionization type is a non-destructive one, in contrast with a classical ionization which occur in case of electron impact ionization (GC-MS), and distinct from the minimal fragmentation that occur in case of MALDI [29,30]

Ion mobility K is the constant that links the drift speed of an ion v_d_ to the electric field intensity E that propels that ion within the IMS cell: v_d_ = K⋅E = l_d_/t_d_ (l_d_ is drift length, and t_d_ is the drift time of a specific ion). Therefore, ion mobility K = v_d_/E = l_d_/(E⋅t_d_). Reduced ion mobility K_0_ is the ion mobility normalized towards temperature and pressure: K_0_ = K⋅(T_ambient_/T_cell_)⋅(P_cell_/P_atmospheric_); K_0_ is widely used as a qualitative parameter in order to characterize a certain compound [1,2].

## Figures and Tables

**Table 1:** Acetic Anhydride AA—formula, chemical and physical properties.

Substance Name	Molecular Formula	Properties	Observations
Acetic anhydride (AA) CAS#: 108-24-7	C_4_H_6_O_3_ (CH_3_CO)_2_O	Vapor pressure: 4 mm Hg (20 °C)	Flammable liquid; Acute toxicity, by inhalation; Acute toxicity, by ingestion; Skin corrosion Conversion: 1 ppm_v_ = 4.25 mg m^−3^
		Molecular Weight: 102.09 g/mol	
		Melting point: −73 °C	
		Boiling point: 138–140 °C	
		Relative density: 1.08 g/cm^3^	
		Vapor density: 3.52 (Air = 1.0)	

**Table 2:** Summary of quantitative results obtained from a photoionization detection (PID) detector and from the Mini IMS instrument in positive ion mode and negative ion mode, respectively (three replicates were used for peak height, in order to calculate standard deviation).

C_AA_ Measured with PID	IMS Data—Positive Ion Mode		IMS Data—Negative Ion Mode	
	Drift Time t_d_ [ms]	Peak Height h [pA]	Drift Time t_d_ [ms]	Peak Height h [pA]
0 ppb_v_	POS RIP 6.28	60.0 ± 2.5	NEG RIP 5.98	95.0 ± 3.6
50 ppb_v_	-	-	PIP#1 6.39 PIP#2 7.10	12.3 ± 0.5 1.5 ± 0.1
100 ppb_v_	-	-	PIP#1 6.41 PIP#2 7.11	24.5 ± 0.8 3.0 ± 0.1
200 ppb_v_	-	-	PIP#1 6.41 PIP#2 7.11	28.3 ± 1.1 4.6 ± 0.1
500 ppb_v_	PIP#1 6.45	4.0 ± 0.1	PIP#1 6.41 PIP#2 7.11	32.5 ± 1.3 7.2 ± 0.2
1000 ppb_v_	PIP#1 6.45	10.0 ± 0.2	PIP#1 6.41 PIP#2 7.11	35.4 ± 1.4 11.8 ± 0.4
1500 ppb_v_	-	-	PIP#1 6.41 PIP#2 7.11	37.9 ± 1.5 15.5 ± 0.4
2000 ppb_v_	PIP#1 6.45	21.0 ± 0.5	-	-
3000 ppb_v_	PIP#1 6.45	27.0 ± 0.7	PIP#1 6.41 PIP#2 7.11	51.0 ± 1.7 17.3 ± 0.6
5000 ppb_v_	PIP#1 6.45	31.0 ± 1.0	PIP#1 6.41 PIP#2 7.11	45.7 ± 1.8 22.5 ± 0.8
6500 ppb_v_	PIP#1 6.45	32.0 ± 1.1	-	-

**Table 3:** Reduced ionic mobilities K_0_ calculated for ions produced by AA.

Operation Mode	Ion Drift Time, t_d_ [ms]	Reduced Ion Mobility^1^, K_0_ [cm^2^ V^−1^ s^−1^]
POSITIVE:	RIP: 6.28	1.94
	PIP #1: 6.45	1.89
	RIP: 5.98	2.04
NEGATIVE:	PIP #1: 6.41	1.90
	PIP #2: 7.11	1.71

^1^ Experimental conditions were: l_d_ = 5.5 cm; E = 400 V cm^−1^; P = 993 mbar; T = 50 °C. Therefore: K_0_ = (1/t_d_)(5.5 × 993 × 293.15 × 10^3^)/(400 × 1013.25 × 323.15) = 12.19/t_d_ [1,2].

**Table 4:** Figures of merit related to IMS detection of AA in negative and positive ion mode.

Ion Mode	LOD [ppb_v_]	LOQ [ppb_v_]	Linear Range [ppb_v_]	Equation	R	S [pA/ppb_v_]
Negative	1.1	3.7	3.7–100	Y = 0.275X + 0.0167	1.000	0.28
Positive	190.0	325.0	325–2000	Y = 0.0113X – 1.5	0.999	0.01

**Table 5:** Specifications of the Mini-IMS ion mobility spectrometer.

Parameter	Specifications
Type of the ion mobility cell	Classic, with stacked rings design (conducting rings alternated with insulating rings).
Ionization source	Radioactive—using the β isotope ^3^H (tritium), with an activity of 300 MBq; tritium was embedded within a stainless steel disc.
IMS cell temperature	ca. 50 °C
Operating pressure	atmospheric pressure (ca. 990 mbar)
IMS cell drift length	l_d_ = 55 mm
IMS cell internal diameter	20 mm
Shutter grid opening time	60 μs
Electric field intensity	E = 400 V cm^−1^
Resolution of the IMS cell	50
Drift gas flow	Purified, dry air at 400 cm^3^ min^−1^; recirculated in a closed-loop pneumatic circuit that contains a filter with 10A molecular sieve.
Sample gas flow	50 cm^3^ min^−1^; provided by a pump operated sequentially
Sampling frequency	Every 15 s (automatic mode)
Inlet gas flow	200 cm^3^ min^−1^; Inlet system uses a sequentially pulsed valve.
Drift gas flow	400 cm^3^ min^−1^; purified air moving inside an internal loop gasflow
Size of the instrument	26.5 × 22 × 14 cm; weight: 3.8 kg
Speed of response	ca. 1 sec after sampling (real time response)
Power supply	Internal rechargeable Li-Ion battery at 19 V d.c. (autonomous mode; min. operation time–ca. 8 h.) or mains (220 V/50 Hz).
Power consumption	6 W
Minimal detectable concentrations MDC	Between 1 ppb_v_ and 100 ppb_v_, depending on the proton affinities of the target analyte (in the positive mode) or on the electron affinities (in the negative mode).
Software	IMS Control Program, ver. 2.209 (IUT GmbH), with spectra deconvolution integrated capability.
